# Identification of REST targets in the *Xenopus tropicalis* genome

**DOI:** 10.1186/s12864-015-1591-4

**Published:** 2015-05-14

**Authors:** Banu Saritas-Yildirim, Christopher P Childers, Christine G Elsik, Elena M Silva

**Affiliations:** Department of Biology, Georgetown University, 411 Regents Hall, Washington, DC 20057 USA; Division of Animal Sciences, University of Missouri, Columbia, MO 65211 USA; Division of Plant Sciences, University of Missouri, Columbia, MO 65211 USA

**Keywords:** REST, NRSE, *Xenopus*, Genome-wide screen, Neurogenesis, Gene silencing

## Abstract

**Background:**

A major role of REST (repressor element-1 silencing transcription factor) is to inhibit the expression of neuronal genes in neural stem cells and non-neuronal cells by binding to a 21 bp consensus sequence and recruiting epigenetic and regulatory cofactors to gene regulatory regions. In neural stem cells, REST silences differentiation-promoting genes to prevent their premature expression and is central to the regulation of neurogenesis and the balance of neural stem cells and neurons.

**Results:**

To understand the role of REST in vertebrate neurogenesis, we performed a genome-wide screen for REST targets in *Xenopus tropicalis*. We identified 742 neuron-restrictive silencer elements (NRSE) associated with 1396 genes that are enriched in neuronal function. Comparative analyses revealed that characteristics of NRSE motifs in frog are similar to those in mammals in terms of the distance to target genes, frequency of motifs and the repertoire of putative target genes. In addition, we identified four F-box ubiquitin ligases as putative REST targets and determined that they are expressed in neuronal tissues during *Xenopus* development.

**Conclusion:**

We identified a conserved core of putative target genes in human, mouse and frog that may be fundamental to REST function in vertebrates. We demonstrate that NRSE sites are associated with both protein-coding genes and lncRNAs in the human genome. Furthermore, we demonstrate that REST binding sites are abundant in low gene-occupancy regions of the human genome but this is not due to an increased association with non-coding RNAs. Our findings identify novel targets of REST and broaden the known mechanism of REST-mediated silencing in neurogenesis.

**Electronic supplementary material:**

The online version of this article (doi:10.1186/s12864-015-1591-4) contains supplementary material, which is available to authorized users.

## Background

The repressor element-1 silencing transcription factor (REST, also called NRSF for neuron-restrictive silencer factor) silences neuronal genes in non-neuronal tissues and in neural stem cells of vertebrates to restrict their expression to neurons and to prevent premature differentiation, respectively [1,2]. REST binds to a conserved 21-bp neuron-restrictive silencing element (NRSE) in the flanking regulatory regions or introns of many neuronal genes [3,4] and recruits the cofactors CoREST [5] and Sin3A [6] to form repressor complexes with histone deactylases [7], histone modifying proteins [8], the methyl-CpG-binding protein MeCP [9,10] and components of the SWI-SNF chromatin remodeling complexes [11]. Together these proteins change the architecture of DNA to heterochromatin and silence target genes [9].

REST is critical for the maintenance of neural stem cells [12-15] and the regulation of neurogenesis. For neuron specific genes to be expressed and neurogenesis to proceed, REST activity is diminished in neural stem cells by two mechanisms; *REST* transcripts are down-regulated [16] and REST protein is targeted for degradation in the proteasome by the beta-tranducin repeat containing/F-box protein with WD40 domain 1 (β-TRCP/Fbxw1 ubiquitin ligase) [17]. Although REST is most commonly reported to silence the expression of protein-coding genes, recent studies suggest that it also regulates non-coding RNAs involved in neurogenesis [18-21]. For example, the neuron specific microRNAs miR-9* and miR-124, important for repression of BAF53a (Brg/Brm association factor 53a) mediated chromatin remodeling and cell cycle exit, are repressed by REST in neural progenitors [20]. REST has also been shown to regulate and interact with long non-coding RNAs (lncRNAs) to control neurogenesis [21-24]. As an example, the nuclear lncRNA, lncRNA_N1, physically interacts with REST during differentiation of hESCs (human embryonic stem cells) to promote neurogenesis [23].

In this study, we performed a screen for REST binding sites and targets in the genome of the diploid amphibian *Xenopus tropicalis*, a model system for genetics and development. Using a degenerate 17 bp sequence derived from 32 bona fide REST targets, we identified 742 NRSE motifs associated with 1396 protein-coding genes. The NRSE distance to genes, number of NRSEs per gene, and the suite of neuronal genes associated with NRSEs in frog are conserved with mouse and human. Through a literature search, we identified which putative target genes are expressed in neuronal tissues and with expression analysis, we verified the restriction of expression of four F-box genes to neuronal tissues. In addition, we found that NRSEs are associated with long non-coding RNAs but not other classes of non-coding RNAs in the human genome.

## Results

### Identification of NRSE sites in the ***Xenopus tropicalis*** genome

**Figure 1:**
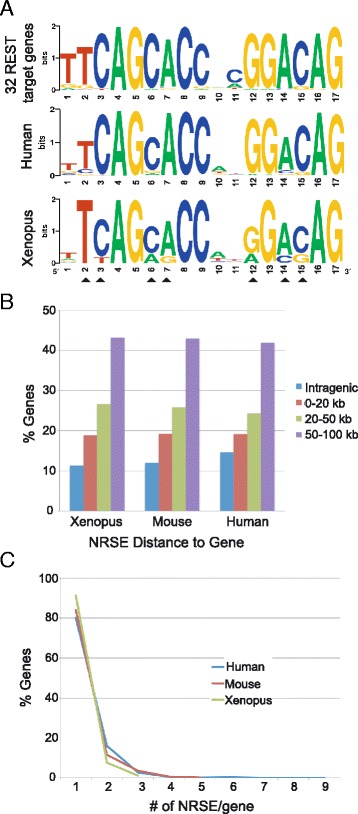
In *X. tropicalis*, human, and mouse, the consensus NRSE motifs share sequence similarity and the majority of the NRSE sites are farther than 20 kb from a gene, with a single copy per gene. **A.** Sequence logos of the consensus NRSE motif derived from 32 bona fide mouse REST target genes ([26], top panel), from 1301 human NRSE motifs ([25], middle panel), and from 742 *X. tropicalis* NRSE motifs (bottom panel). Arrowheads mark the differences between the *Xenopus* and human consensus motifs. **B.** In all three vertebrate genomes, ~ 65% of the motifs are located farther than 20 kb from a gene. **C.** 80% to 90% of the putative REST target genes in three organisms have a single NRSE motif within 100 kb of a gene.

To identify NRSE sites in the *X. tropicalis* genome, we performed an *in silico* screen of the genome for a 17 bp degenerate NRSE consensus motif (NTYAGMRCC**NN**RGMSAG) generated from 32 *bona fide* REST target genes in human, rodents, and chicken [25,26]. The consensus NRSE motif has two highly conserved regions (5′ half and 3′ half) separated by a linker region that consists of 2 poorly conserved nucleotides (in bold) [27]. The consensus NRSE motif does not include the lesser-conserved 4 nucleotides at the 3′ end found in the canonical 21 bp consensus [25]. We retrieved all NRSE sites and annotated each site based on genomic location (Additional file 1: Table S1). We found 742 NRSE sites with 340 permutations in the *Xenopus* genome. The consensus sequence of the *Xenopus* NRSE varies slightly from that of human. For example, whereas nucleotide A is predominant at position 7 in the human NRSE consensus, both A and G are in high occupancy at this position in *Xenopus* (chi-square test, p < 1.0E-6) (Figure 1A).

REST binds to NRSEs in intergenic and intragenic regions [26]. To determine the location of NRSE motifs with respect to genes, we used a cut-off distance of NRSE to genes of 100 kb to facilitate the comparison of our screen to one in mammals using the same degenerate sequence [25]. In human, mouse, and *Xenopus,* the majority of the NRSEs are located in the flanking regions of genes at a distance greater than 20 kb (Figure 1B). Next, we identified the number of motifs within 100 kb of a gene (Figure 1C). Common in both mammals and *Xenopus*, 80-90% of putative REST target genes are associated with a single motif; less than 10% of the targets genes are associated with two or more motifs (Figure 1C).

To determine the proportion of *Xenopus* NRSEs that are directly orthologous to the human NRSEs, we retrieved pairwise alignments of human (GRCh37) and *X. tropicalis* (JGI 4.2) genomes generated by genome-wide comparison using Blastz [28] from the UCSC genome browser [29] and analyzed the homologous sequences for the presence of NRSE sites. With a chain score cutoff of 5000, the summed length of homologous *Xenopus* regions in the pairwise alignments was 657,812,008 bp, or 2% of the *Xenopus* genome. We identified 85 homologous regions with sizes ranging from 42–2667 bp that have NRSE motifs in the *Xenopus* homolog. However, only 12 of these 85 have an NRSE motif in the human homolog. Thus, 11.5% of the *Xenopus* NRSE sites are in regions of the genome with homology to the human genome, and only 14% of those regions have NRSE sites in both species. The small number of homologous regions with NRSEs is likely due to the low level of homology in non-coding regions between frogs and humans.

In total, we demonstrated that the NRSE consensus, distance from gene, and the number of motifs within 100 kb of a gene are similar in *Xenopus*, mouse, and human. However, the locations of NRSE motifs in homologous regions are not conserved among frogs and humans.

### Species-specific features of ***X. tropicalis*** NRSEs

**Figure 2:**
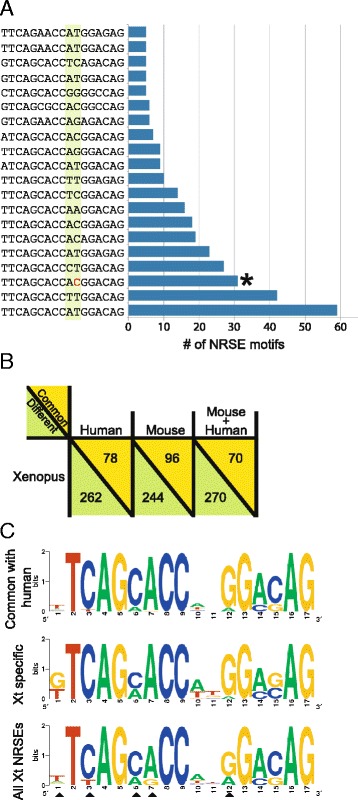
The species-specific features of *X. tropicalis* NRSEs. **A.** The most abundant 20 motifs and copy number in the *X. tropicalis* genome. The asterisk marks the most abundant motif in the humans genome. The green highlight marks the linker region (positions 10 and 11) of the NRSE motif. The red “C” is the only difference between the most abundant NRSE motifs in *Xenopus* and humans. **B.** The number of NRSE motif permutations in common between the three vertebrate genomes. **C.** The consensus motifs derived from the 78 NRSEs in common between humans and *X. tropicalis*, and the 236 *X. tropicalis* specific motifs. Arrowheads show the deviations from the *X. tropicalis* consensus derived from all motifs.

The *Xenopus* consensus motif deviates slightly from that of human and mouse. To determine where these differences lie, we first determined the frequency of each NRSE motif permutation in the genome. The degenerate NRSE sequence used to search the genome can produce 4076 permutations; however, only 340 permutations were represented in the *X. tropicalis* genome. The 340 motif permutations in 742 unique genomic loci had varying frequencies in the genome. Nearly 200 motif permutations are present only once in the genome while the most abundant motif is replicated 59 times (Figure 2A). The most common motif in the human genome is the third most common in the *X. tropicalis* genome. The only difference between these two motifs is a single nucleotide change in the linker region; T at position 11 of the *Xenopus* motif and C in human. It has been shown that the length of the linker region, but not the identity of the nucleotides, is important for the function of REST [27]. Therefore, the differences we found in the linker region are not likely to have an effect on the binding efficiency and gene silencing capacity of REST.

To identify the *Xenopus*-specific motifs, we compared the *Xenopus* NRSEs to the human and mouse motifs. Among the 340 *Xenopus* NRSE motif permutations, only 70 (20.5%) are in all three genomes (Figure 2B). 22.9% of the *Xenopus* NRSE motifs are found in the human genome with 8 motifs exclusively shared between the two, and 28.2% of the *Xenopus* NRSEs are in the mouse genome with 26 motifs shared exclusively. Thus, approximately 70% (236 out of 340 motifs) of the *X. tropicalis* motifs are unique to the *Xenopus* genome (Figure 2B).

We generated a *X. tropicalis* specific consensus NRSE from 236 motifs and a *Xenopus*-human consensus motif from the 78 motifs shared between human and *Xenopus* (Figure 2C). There are significant shifts in abundance of nucleotides between *Xenopus* and human at positions 6 in the 5′-half of the motif, at position 10 in the linker region, and at position 15 in the 3′-half (chi-square test, p < 1.0E-6). These variations contributed to the deviation of the *Xenopus* consensus from the mammalian one and the generation of a species-specific version of the NRSE consensus motif.

### 17-24% of NRSE motifs in the human, mouse and frog genomes are located in regions devoid of protein-coding genes

**Figure 3:**
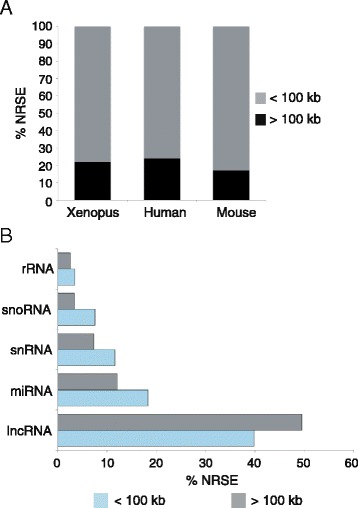
Human NRSEs are associated with lncRNAs genome-wide but not in gene-distant regions. **A.** 17-24% of NRSE motifs in the human, mouse and frog genomes are located in gene-distant regions (GD). **B.** In the human genome, NRSEs within 100 kb of ncRNAs are associated with lncRNAs (chi-square, p = 0.00102547). In the figure, <100 kb denotes the NRSEs that are within 100 kb of a protein-coding gene and >100 kb is for NRSEs that are located in GD.

We found that 22% of all *Xenopus* NRSE sites are located in what we call “gene-distant regions” (GD), which are regions greater than 100 kb from protein-coding genes (Figure 3A). To investigate the function of the NRSEs in GD, we first compared the GD consensus motif to that of NRSEs located within 100 kb of a protein coding gene. There was no difference in consensus motif sequences distinguishing the motifs in low gene occupancy regions as non-functional (Additional file 2: Figure S1). To test whether these motifs may be involved in the regulation of non-coding RNAs (ncRNAs), we analyzed the relationship of the NRSEs with ncRNAs in the human genome since these are better annotated than *Xenopus* ncRNAs and there are fewer gaps than in the *Xenopus* genome. Assembly gaps increase error in estimations of distances between NRSEs and genes. Using our screen to retrieve NRSEs in the human genome, we identified 4058 motifs. We eliminated 12 of the 4058 NRSEs, because they are located on unassigned scaffolds, and therefore prone to errors in distance calculations. The distribution of distances from 4046 NRSEs to the nearest protein-coding genes ranges from 0 to 2,070,000 kb, with a mean of 118,800 kb and a median of only 10,470 kb. Of the 4046 NRSEs, 980 (24%) are located in GD. Of the NRSEs in gene-distant regions, 597 (61%) are located within 100 kb of ncRNAs, while 2408 (51%) of all NRSEs are associated with ncRNAs genome-wide. Of the ncRNA classes provided by Ensembl (lncRNA, miRNA, rRNA, snRNA, snoRNA), NRSEs are associated most frequently with lncRNAs; 485 (49%) of the NRSEs in GD are located within 100 kb of lncRNA genes, while 1610 (39%) of all NRSEs are associated with lncRNA genome-wide (Figure 3B).

We performed chi-square tests to determine whether the numbers of NRSEs associated with ncRNA and protein-coding genes were greater than expected by chance. Expected counts were estimated by shuffling the coordinates of the human NRSE motifs. To test for significant associations of NRSE with the five ncRNA classes (lncRNA, miRNA, rRNA, snRNA, snoRNA) and protein-coding genes on a genome-wide basis, the coordinates of the 4046 NRSE were shuffled within each chromosome 1000 times such that shuffled NRSE coordinates were not allowed to fall within assembly gaps. For each class of ncRNA gene, the number of NRSEs within 100 kb of a gene was determined for each shuffled dataset, and the average numbers of 1000 datasets were used as expected counts in chi-square tests (Additional file 3: Table S3). We found that NRSE sites genome-wide are associated with lncRNAs more often than expected by chance (chi-square test, p = 0.00102), but there are no significant associations of NRSE with any other class of ncRNA. Not surprisingly, NRSEs are found within 100 kb of protein-coding genes more often than expected by chance (p = 0.0008). The significant association of NRSEs with lncRNAs on a genome-wide basis may have been due to the frequency of lncRNAs in close proximity to protein-coding genes.

We next evaluated the subset of 980 NRSEs that are not located within 100 kb of protein-coding genes (i.e. within GD). Again, we shuffled the NRSE coordinates within each chromosome 1000 times. In addition to disallowing shuffled NRSE coordinates to fall within assembly gaps, we did not allow shuffled NRSEs to fall within 100 kb of protein-coding genes. Using the average counts from 1000 datasets to estimate numbers of NRSE within 100 kb of ncRNAs, the chi-square tests did not show a significant association of NRSE with any class of ncRNA given the NRSE were located in GD (Additional file 3: Table S3).

### A conserved group of putative REST target genes is enriched in neuronal development and function

We identified 1396 unique protein-coding genes in the *X. tropicalis* genome that are within 100 kb of one or more NRSE motifs (Additional file 1: Table S1).

**Figure 4:**
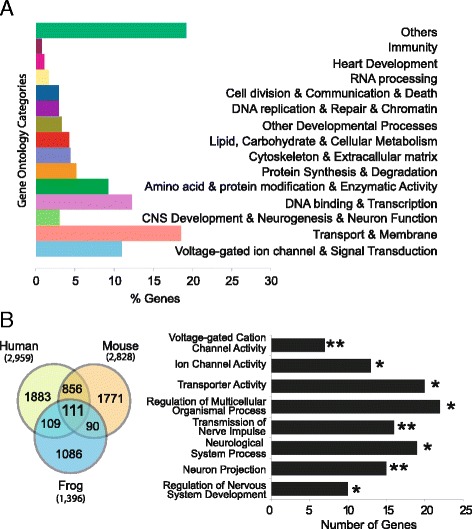
Gene Ontology classification of putative NRSE target genes. **A.** Genes were linked to 14 functional groups or “others” subgroup based on GO descriptions. **B.** The 111 common NRSE target genes in human, mouse, and frog genomes are enriched in neuronal functions. All categories are statistically significant with single and double asterisks showing p-values <0.05 and <0.01, respectively.

To distinguish functional groups within the gene list, we retrieved Gene Ontology (GO) terms for *X. tropicalis* genes using Ensembl Biomart (Additional file 4: Table S4). The GO terms were further categorized into fourteen general functional groups (Figure 4A).

To determine whether there was a conserved core of REST target genes in vertebrates, we first identified the genes in common within 100 kb of the 12 conserved NRSEs in the homologous regions of the *Xenopus* and human genomes. We identified 22 genes within 100 kb of the 12 conserved NRSEs in the *Xenopus* genome (Additional file 5: Table S2) and 64 genes in the human genome with 9 genes in common (*bdnf, dnajc5b, mtmr14, pou4f1, rnf219, setd5, slc4a1, trim55, and ubtf*). Gene ontology analysis showed that of the 9 common genes, bdnf [30-32], pou4fl [33,34], rnf219 [35] and setd5 [36-38] have functions in neurogenesis and disease of the nervous system.

We broadened the search for a conserved set of vertebrate target genes to include targets shared with mouse, human and *Xenopus*. We first identified all of the genes within 100 kb of an NRSE motif in human and mouse (Figure 4B). Only 111 (8%) of the *X. tropicalis* genes within 100 kb of an NRSE motif (with average distance of 47,954 bp and median of 46,385 bp) are also putative REST targets in mouse and human based on comparison of gene names (Additional file 6: Table S5). The list of shared genes, categorized into functional GO groups using g: Profiler software (Table 1), are significantly enriched in neuronal functions, including voltage-gated cation channel activity, transmission of nerve pulse, neuron projection, and regulation of nervous system development (Figure 4B). These genes may comprise a core group of protein-coding genes that are central to REST-meditated gene silencing.

**Table 1:** **Gene Ontology classification of conserved core 111 common genes using g:profiler**

**GO term definition**	**GO term**	**No. of genes**	**P-value**
Regulation of Nervous System Development	GO:0051960	10	4.32E-02
Regulation of Multicellular Organismal Process	GO:0035637	15	5.52E-04
Neurological System Process	GO:0050877	19	1.63E-02
Transmission of Nerve Impulse	GO:0019226	16	8.55E-04
Neuron Projection	GO:0043005	22	2.95E-02
Transporter Activity	GO:0005215	20	1.01E-02
Ion Channel Activity	GO:0005216	13	3.29E-04
Voltage-gated Cation Channel Activity	GO:0022843	7	2.96E-02

### In silico validation of the NRSE screen

The *Xenopus* NRSE screen successfully identified 25 of 32 bona fide REST target genes [26]. We also used two *in silico* approaches to validate the screen. First, we searched Xenbase, a publicly available gene expression database for *X. tropicalis* and *X. laevis* [39], for the spatial expression profile of each gene with an NRSE motif as determined by whole mount *in situ* hybridizations. Neuronal– and heart-restricted genes [40] including those expressed in the neural plate, neural tube, brain, spinal cord, and eyes (but not neural progenitors or stem cells) were considered true positives. Although REST splice variants are also expressed in thymus, kidney, testis, lung, spleen, and muscle [13], the experimental evidence for REST function in these peripheral tissues is lacking. Therefore, we did not include gene expression in such peripheral tissues to indicate regulation by REST. Expression elsewhere was considered a false positive. Out of 1396 genes, only 206 had *in situ* expression data in Xenbase (Table 2). Among 206 genes, expression of 141 genes (68%) is restricted to neuronal and heart tissues. Of these 141 genes, 16 are among the 111 putative REST target genes common in human, mouse, and frog genomes.

**Table 2:** ***In silico*** **validation of the** ***Xenopus*** **NRSE screen with** ***in situ*** **hybridization and REST CHIP-seq. Putative REST target genes were categorized into neuronal/heart or non-neuronal/non-heart expression based on** ***in situ*** **hybridization data collected from Xenbase**

***in situ*** **hybridization**		**REST CHIP-Seq**
**Neuronal/Heart Expression 141 genes**	**Non-neuronal/non-heart Expression**	**57 genes**
**Bsx**, arl6ip1, cox5a, ag1, **cacna1h**, adarb1, ahctf1, asb8, churc1, cpsf2, cpeb1, dact1, adprh, bmp7.1, bsn, cldn5, asap1, arid4a, cdca8, cdk1, calu, colec11, arl8a, bri3, admp, cdh12, arx, celf2, cdc45, **cacna1a**, col18a1, CACNA1C, calb1, clasp1, copg, crb2, dlx4, dmrta2, dnal4, dvl1, egr1, eif4h, **elavl4**, **elk1**, ephb1, erbb4, ern2, esr10, esyt2, ext1, fam54b, fgf12, frzb2, fzd3, gabbr2, gabra3, gabra5, gbx2.1, gbx2.2, gcat, gdf11, gdi1, gfi1, gjb1, glmn, gpr84, hes4, hnf4a, hnrnpa1, hpcal1, id2, igfbp4, insm1, ism1, kaz, klf11, **klhdc4**, **lhx2**, **lhx3**, **lhx5**, limk1, **mef2d**, mnt, mnx1, myl7, myo1c, myo1d, nbl1, **ncoa5**, **nefm**, neurod1, neurog3, nol10, nr2f2, pcdh10, pcmtd1, pla2g7, plxnb1, pnhd, **pou4f1**, prph, rab34, rab7a, rasip1, rax, rbm38, rgs20, rhbdd3, **ric8a**, rps3, scn2a, **scn3a**, selt, sema3a, siah2, slc32a1, slc3a2, slit1, smad4, smarca4, snai1, sox14, sphk1, spry2, srsf5, srsf6, supt6h, suv420h1, tbx5, tcea1, tmub2, tpm4, tubgcp4, vamp1, **vav2**, wdr5, wdr73, wdr74, wnt16, wnt3a, wnt9b	Alb, alg3, amy2b, anxa4, arf1, armc4, baiap2l1, bsg, col1a1, CREB3L2, ctsc, cxcr7, dazap1, dcdc2, fgf14, fuz, gamt, gfpt1, gorasp2, grhl1, gstp1, igsf9b, impdh1, iqgap1, iqgap2, itga8, klf5, krt5.7, laptm4a, ldlrap1, mmp9, mst1r, myos, nbn, ndufaf3, nodal, nom1, odc1, pcdh8, ppp1r3c.1, rab18, rab8a, rnd1, sept2, sept9, sfrp5, sgk2, snd1, sox17b.1, sox2, trappc2, tspan7, tspan8, ttll4, upk1b, ventx1.2, ventx2.1, ventx2.2, ypel5, zdhhc1, zdhhc4, zfpm1	Angptl6, ap1s1, ap3b2, brsk2, **bsx**, **cacna1a**, cacna1h, cacna2d2, cdh22, cdb4, chd5, chat, cpsf3, cyp27b1, decr1, ebf1, fgf14, glra1, grin1, hes3, icmt, kcnc3, kcnh4, kndc1, **lhx3, lhx5**, lin37, march11, **nefm**, neto1, nr2f1, nup133, ogdh, olfm3, pafah1b1, pcgf6, pipox, plbd2, plbd2, **pou4f1**, ptk2b, pusl1, qsox2, rdh8, ric8b, rnf219, sdsl, slc35f4, slc4a1, slc4a1ap, slc5a11, syt4, taf5, vwc2l, xkr7, zcchc14, rasgrf1

Out of 1,396 genes, 206 had expression data, 141 (68%) of which had expression in neuronal/heart tissues. REST CHIP-Seq targets were retrieved from [41-43]. The bold emphasis indicates the 16 genes among the 111 putative REST target genes common in human, mouse, and frog genomes.

The published REST Chip-seq data in human [41-43] was also used to validate the putative REST targets genes conserved among *Xenopus*, mouse and human in our *in silico* screen. The REST Chip-seq analysis identified 57 (51%) of the 111 putative REST target genes shared between mouse, human and frog indicating these as true targets of REST (Table 2). Thus, the *in silico* validation of our screen suggests that we successfully identified putative REST target genes in the *Xenopus* genome.

### In vivo validation of the NRSE screen

**Figure 5:**
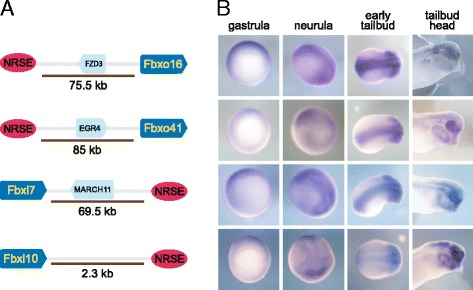
*In vivo* validation of the *Xenopus* NRSE screen. Four F-box ubiquitin ligases identified in the NRSE screen are expressed in the neuronal tissues of *X. tropicalis.* **A.** The genomic localization of the genes with respect to NRSE motifs and **B.** their mRNA expression hybridization during *X. tropicalis* development. Gastrula embryos are ventral view with dorsal to the top. Neurula embryos are dorso-lateral view with anterior to the right. Early tailbud embryos are dorsal view with anterior to the right. Tailbud heads are lateral view with anterior to the right. The arrows point the direction of genes. Intervening genes are in turquoise. Cartoons are not to scale.

The link between REST and protein degradation has not been well established although both have fundamental roles in neurogenesis [44]. Towards understanding the regulatory relationship between REST and protein degradation, we studied 4 F-box genes *fbxo16*, *fbxo41*, *fbxl7*, and *fbxl10*, identified in our screen. F-box proteins are the E3 ligase components of RING type ubiquitin ligases [45]. The NRSE motifs associated with the F-box genes are located upstream or downstream of the genes at a distance >50 kb except for Fbxl10, which has an NRSE within 2.3 kb of the gene start (Figure 5A). There is an intervening gene between *fbxo16*, *fbxo41*, and *fbxl7* and the NRSE. To determine if these genes are restricted to neuronal tissues, we analyzed their expression in *X. tropicalis* embryos using *in situ* hybridization. All four genes are expressed in the developing embryo from gastrula to tailbud stages (Figure 5B). In early gastrulae (st 10.5), the genes are weakly expressed in the ectoderm with greater expression in the dorsal neuroectoderm. However, expression increases at the neurula stage (st. 17) and all genes are primarily expressed in the neural tube. Whereas *fbxo16*, *fbxo41*, and *fbxl7* are pan neural, *fbxl10* is localized to the anterior-most and posterior-most regions of the neural plate. At early tailbud stages (st. 25 & st. 30), all genes are expressed in the brain with *fbxo41*, *fbxl7* and *fbxl10* also expressed in the eyes and branchial arches. In *Xenopus*, REST is maternal and uniformly expressed in the ectoderm during gastrula stages (Additional file 7: Figure S2). However, the expression is diffuse in the neurula embryo including the neural folds and then later restricted to the brain and spinal cord in tailbud stages (Additional file 7: Figure S2 and [2]). At the cellular level, REST is expressed in neural progenitors and stem cells but excluded from differentiating and mature neurons [9,13]. Our expression analysis confirmed the NRSE screen and showed that the expression of four putative F-box genes is localized to neural tissues during *Xenopus* development.

**Figure 6:**
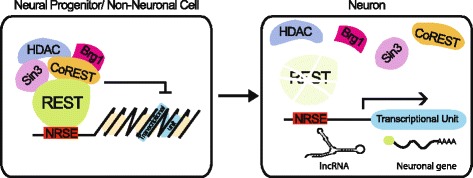
REST mediated repression of coding and non-coding gene expression. In neural progenitors and non-neuronal cells, REST facilitates silencing of expression by binding to highly conserved NRSE elements and recruiting co-repressors and chromatin remodeling agent to convert the topology of the local DNA to heterochromatin. During neurogenesis, REST transcription is down regulated and the protein is degraded. The absence of REST in neuronal cells allows the expression of neuron specific protein-coding genes and non-coding RNAs including micro and long non-coding RNAs.

## Discussion

With a genome-wide screen, we identified 742 NRSE motifs associated with 1396 protein-coding genes (within 100 kb) in the *X. tropicalis* genome. We compared the data of our screen in *Xenopus* to that of screens in human and mouse [25]. The three vertebrate data sets have similar features in terms of the number of NRSE motifs associated with genes, their distance to genes, and the GO classification of genes with NRSEs. Furthermore, we identified four F-box ubiquitin ligases as putative REST target genes in *Xenopus* with expression restricted to the neuronal tissues in developing embryos. Our analysis, therefore, identified a new regulatory relationship between REST and the components of protein degradation machinery (Figure 6).

Screening the *Xenopus* genome, we identified the majority of the previously identified *bona fide* human REST target genes including NaV1.2 [26], one of the first REST target genes identified. However, we failed to identify SCG10 (also known as STMN2), another well-studied REST target gene [1]. In fact, no NRSE motif was present on the scaffold containing SCG10. To investigate the possibility that SCG10 is regulated by an unconventional NRSE motif, we searched the scaffold for partial (i.e. 5′-half or 3′-half) and bipartite NRSE motifs (i.e. the NRSE motifs with 5′ and 3′ halves separated by more than 2 linker nucleotides) [27]. We identified 17 5′-half and 262 3′-half motifs within 100 kb of SCG10 but no bipartite motifs. This suggests that the regulation of SCG10 expression in *Xenopus* either involves an unconventional regulatory mechanism such as REST binding to partial motifs or is regulated independent of REST.

Our genome-wide analysis was based on a conserved consensus NRSE motif that allowed degeneracy at certain positions in the motif. However, it did not identify non-traditional NRSE motifs such as bipartite, partial or species-specific motifs, which could only be identified through Chip-seq analysis. However, our analysis using the conserved NRSE motif did identify the conserved target genes among vertebrates.

It is surprising that only 8% of NRSE target genes are conserved among the three vertebrate genomes. This could be due to the fact that REST is able to bind degenerate NRSE motifs. In fact, the NRSE motifs in functionally validated REST target genes show differences in sequence but yet satisfy REST binding [26]. The flexibility in binding may allow the emergence of new sites through nucleotide substitution, and hence, the recognition of new genes. As expected, we identified a larger number of conserved NRSE target genes among mammals than across the greater evolutionary distance of the three vertebrates. We found more than 30% of NRSE target genes to be conserved between human and mouse but only 8% were conserved among in human, mouse, and frog (Figure 4B). The more conserved genes might have undergone high selective constraint against changes in the REST sites, and are functionally essential. Lesser-conserved genes with relaxed constraints may have allowed the emergence of new NRSE sites.

While our analysis did not reveal a significant association of NRSEs with lncRNA in gene-distant regions (GD), we did find NRSEs to be associated with lncRNA genome-wide. Our analysis could not exclude the possibility that the significant association of NRSEs with lncRNA was due to the close proximity of lncRNA and protein-coding genes. However, evidence for the regulation of lncRNAs by REST continues to emerge [21-24].

## Conclusions

With a screen for REST binding sites in the *X. tropicalis* genome and a comparison of the characteristics of these binding sites in mouse, human and frog, we determined that NRSEs are most commonly located greater than 20 kb from a protein-coding gene in single copies, that there is a *Xenopus*-specific consensus, that only 20.5% of the Xenopus NRSEs are also in mouse and human and that there is a conserved core of putative REST target genes enriched in neuronal function in these three vertebrates. We also identified four F-box proteins as putative target proteins thereby linking ubiquitin-mediated degradation with regulation of neurogenesis.

## Methods

### In silico detection and characterization of NRSE binding sites

A genome-wide search for NRSE binding sites in the *X. tropicalis* genome (genome assembly version JGI 4.2 [46]) was performed as described [25]. A unique identifier was given to each NRSE motifs based on the genomic localization. To facilitate the comparison to those screens in mammalians [25], similar criteria and settings were used in analysis, including the same consensus NRSE sequence, with human genome assembly GRCh37 [47] and mouse genome assembly MGSCv37 [48]. Motif logos were generated using WebLogo (http://weblogo.berkeley.edu/logo.cgi) [49].

### Identification of homologous regions in the human genome

To identify regions in the human chromosome that were homologous to *Xenopus* regions containing NRSE motifs, pairwise genome alignments between human genome assembly GRCh37 and *X. tropicalis* genome assembly JGI4.2 were downloaded from the UCSC Genome Browser web site (http://hgdownload.soe.ucsc.edu/goldenPath/xenTro3/vsHg19/xenTro3.hg19.all.chain.gz). We performed our NRSE screen on sequences provided in the chain file to determine whether regions in the *Xenopus* genome with NRSE motifs had homologous regions in the human genome with NRSE motifs.

### Annotation of genes within 100 kb of NRSE

*Xenopus*, human, and mouse annotations for both coding- and non-coding gene sets were retrieved from Ensembl which uses GENCODE, a merge of the automatic annotation from Ensembl and the manually curated annotation from Havana. *X. tropicalis* genes with descriptions, genomic location, and Gene Ontology (GO) terms were downloaded from Ensembl Biomart [50]. The genes common in the human, mouse, and frog NRSE screens were identified through the comparison of gene names.

A higher ordering of GO terms was achieved by manual assignment to 14 functional groups (Voltage-gated ion channel and signal transduction, Transport and membrane, CNS development, neurogenesis and neuron function, DNA binding and transcription, Amino acid, protein modification and enzymatic activity, Protein synthesis and degradation, Cytoskeleton and extracellular matrix, Lipid, carbohydate and cellular metabolism, DNA replication, repair and chromatin, Cell division, communication and death, RNA processing, Heart development, Other developmental processes, Immunity). For the common genes in the human, mouse, and frog screens, GO classification was done with g: Profiler (http://biit.cs.ut.ee/gprofiler/) [51] using default settings.

### Analysis of NRSE association with ncRNA

We used the human genome assembly GRCh37 and Ensembl human annotation release GRCh37.75 to investigate potential associations of NRSE with ncRNA. The annotation file in gtf format was retrieved from the Ensembl ftp site (ftp.ensembl.org). We parsed the gtf-formatted file into separate files for protein-coding genes, lncRNA, miRNA, rRNA, snoRNA and siRNA, keeping only the gene features so that genes with multiple transcripts would not be counted more than once. We used the intersectBed program from the BedTools package [52] to identify motifs that fell within or outside the ranges of interest. In order to identify motifs located within or further than 100 kb of a gene, we modified gtf files to extend gene coordinates 100 kb in each direction. For example, to identify motifs further than 100 kb from protein-coding genes (i.e. gene-distant regions or GD), we used intersectBed with the –v option (show features that do not overlap) to output NRSE motif coordinates that do not overlap protein-coding gene coordinates which had been extended by 100 kb. We then used intersectBed with the –u option (list each feature in set A once if it overlaps set B) to intersect the coordinates of the previous output with lncRNA coordinates which had been extended 100 kb, to identify NRSE that were greater than 100 kb from protein-coding genes but within 100 kb of the lncRNA. We repeated this for each ncRNA class, and we performed similar intersections with the entire NRSE dataset to investigate genome-wide associations.

For chi-square tests to determine whether the numbers of NRSE within 100 kb of ncRNA and protein-coding genes were greater than expected by chance, we estimated expected counts by shuffling the coordinates of the human NRSE motifs using the shuffle program from the BedTools suite. First, we used the Table Browser available from UCSC Genome Browser to create a file of genome assembly gaps, and used it with the –excl option of the shuffle program to exclude gaps from possible shuffled locations. The –chrom option was used so that locations were permuted within each chromosome instead of randomly in the genome. Shuffling was performed 1000 times each on the total human NRSE dataset (4046 motifs with chromosome coordinates) and the dataset of 980 NRSE in GD, so that we could perform separate chi-square tests for each dataset. For each dataset, 1000 shuffled NRSE gtf files were used as input to intersectBed with the –u option to perform intersections with coordinates of different ncRNA classes that had been extended by 100 kb in each direction. In the case of the gene desert dataset, shuffled motif locations were excluded from within 100 kb of protein-coding genes (i.e. they had to remain in GD). This resulted in 1000 genome-wide and 1000 gene desert counts for NRSE located within 100 kb of each class of ncRNA. We used the average of 1000 counts as the expected count for each chi-square test.

### Whole mount in situ hybridization

Whole-mount in situ hybridization (WISH) was performed as described [53,54] with the following modifications: pre-hybridization treatment was extended to overnight and an additional 1X SSC wash (15 min, room temperature) was added. *X. tropicalis* embryos were gifts from M. Khokha (Yale U., Connecticut). *X. tropicalis* Fbxl7, Fbxl10, Fbxo16, and Fbxo41 clones were gifts from R. Harland (U. of California, Berkley). The riboprobes were digoxigenin-labeled.

### Ethics statement

This study was carried out in strict accordance with the recommendations in the NRC Guide for the Care and Use of Laboratory Animals. The protocol was approved by the Georgetown University Animal Care and Use Committee (GUACUC, Protocol: 13–016). Euthanasia was performed under the American veterinary medical association guidelines, and all efforts were made to minimize suffering.

### Availability of supporting data

The data sets supporting the results of this article are included within the article and its additional files.

## Additional files

Additional file 1: Table S1.
*X. tropicalis* protein-coding genes within 100 kb of one or more NRSE motif. Additional file 2: Figure S1. Consensus sequence logos for NRSE motifs associated with protein-coding genes and in gene-distant regions are similar. NRSE motifs associated with protein-coding genes were located ≤ 100 kb distance. The NRSEs in GD were located > 100 kb from a protein-coding gene. Additional file 3: Table S3. Chi-square tests for association of NRSEs with ncRNAs in the entire human genome and in gene-distant regions based in shuffling NRSE coordinates. Additional file 4: Table S4. GO terms for *X. tropicalis* protein-coding genes within 100 kb of an NRSE motif. Additional file 5: Table S2. The genes associated with 12 NRSE motifs identified in homologous regions of human and *X. tropicalis* genomes. Additional file 6: Table S5. REST target genes conserved in mouse, human and frog. Additional file 7: Figure S2. The expression of *REST* during *Xenopus laevis* development using *in situ* hyridization. In the egg, animal pole is to the top. Gastrula embryos are ventral view with dorsal to the top. Tailbud embryo is lateral view with anterior to the left.

## Abbreviations

REST: Repressor element 1 silencing transcription factor
NRSE: Neuron-restrictive silencing element
ncRNA: Non-coding RNA
lncRNA: Long-non coding RNA
miRNA: MicroRNA
rRNA: Ribosomal RNA
snRNA: Small nuclear RNA
snoRNA: Small nucleolar RNA
MeCP: Methyl-CpG-binding protein
βTRC: Beta-transducin repeat containing protein
Fbxw: F-box protein with WD40 domain
Fbxl: F-box protein with leucine rich repeat
Fbxo: F-box protein with domain other than WD40 or leucine rich repeat
BAF53a: Brg/Brm associated factor 53a
hESC: Human embryonic stem cell
GD: Gene-distant regions
GO: Gene ontology
WISH: Whole-mount in situ hybridization
