# Supplementary figures and images for: Identification of REST targets in the Xenopus tropicalis genome
Source: BMC Genomics. 2015 May 14;16(1):380. doi: 10.1186/s12864-015-1591-4 (PMC4430910; doi:10.1186/s12864-015-1591-4)

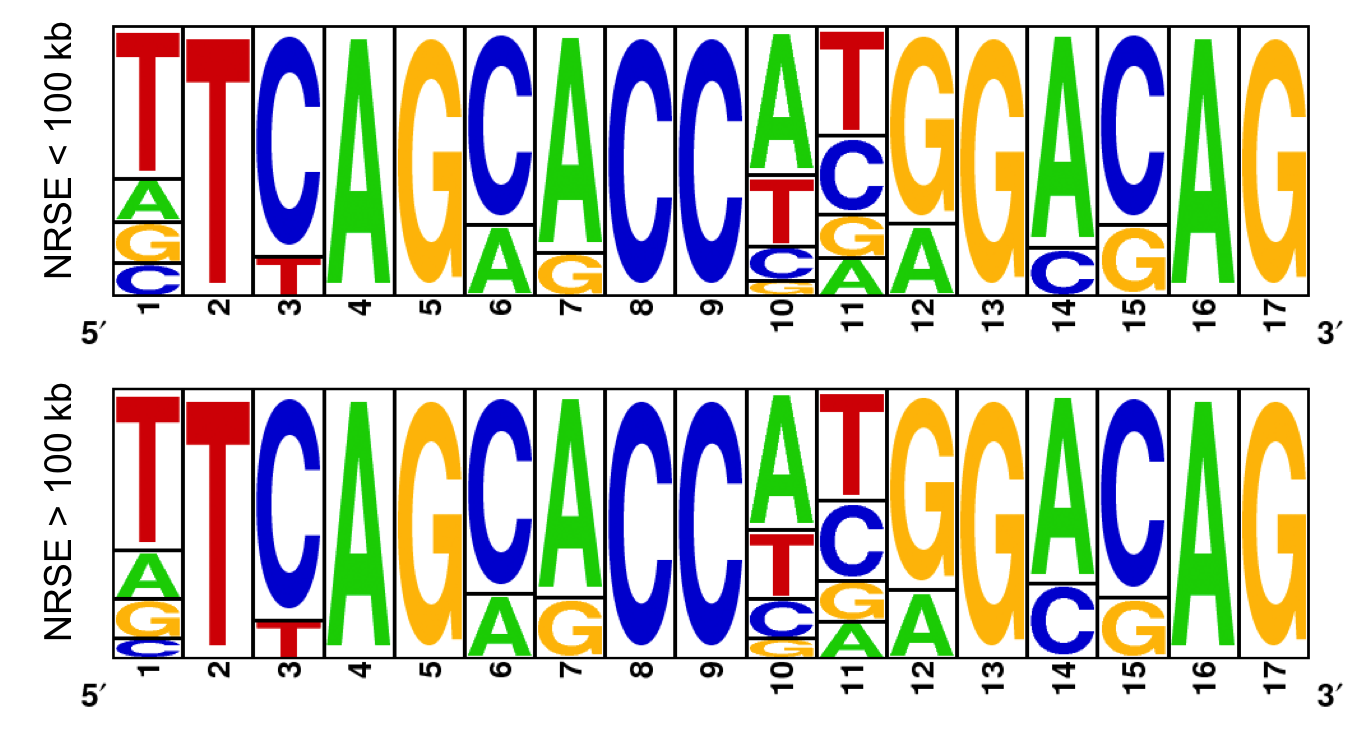

Supplement: Additional file 6: Table S5. — REST target genes conserved in mouse, human and frog. [file 12864_2015_1591_MOESM6_ESM.png]
